# Transformation gap from research findings to large-scale commercialized products in microfluidic field

**DOI:** 10.1016/j.mtbio.2024.101373

**Published:** 2024-11-29

**Authors:** Yuqi Ma, Xiaoyi Sun, Ziwei Cai, Mengjing Tu, Yugang Wang, Qi Ouyang, Xueqing Yan, Gaoshan Jing, Gen Yang

**Affiliations:** a State Key Laboratory of Nuclear Physics and Technology, School of Physics, Peking University, Beijing, 100871, China; b Wenzhou Institute, University of Chinese Academy of Sciences, Wenzhou, 352001, China; c Center for Quantitative Biology, Peking University, Beijing, 100871, China; d Institute of Microelectronics, Chinese Academy of Sciences (CAS), Beijing, 100029, China

**Keywords:** Microfluidics, R&D, Commercialization, PDMS, Thermoplastic

## Abstract

The field of microfluidics has experienced rapid growth in the last several decades, yet it isn't considered to be a large industry comparable to semiconductor and consumer electronics. In this review, we analyzed the entire process of the transformation from research findings to commercialized products in microfluidics, as well as the significant gap during the whole developing process between microchip fabrication in R&D and large-scale production in the industry. We elaborated in detail on various materials in the microfluidics industry, including silicon, glass, PDMS, and thermoplastics, discussing their characteristics, production processes, and existing products. Despite challenges hindering the large-scale commercialization of microfluidic chips, ongoing advancements and applications are expected to integrate microfluidic technology into everyday life, transforming it into a commercially viable field with substantial potential and promising prospects.

## Highlights

• Discussion of the significant transformation gap from R&D to commercialization in microfluidic field.
• Detailed overview of various materials utilized in microfluidic industry.
• Presentation of representative examples of commercialized products in microfluidic industry.

## Graphical abstract

**Figure:**
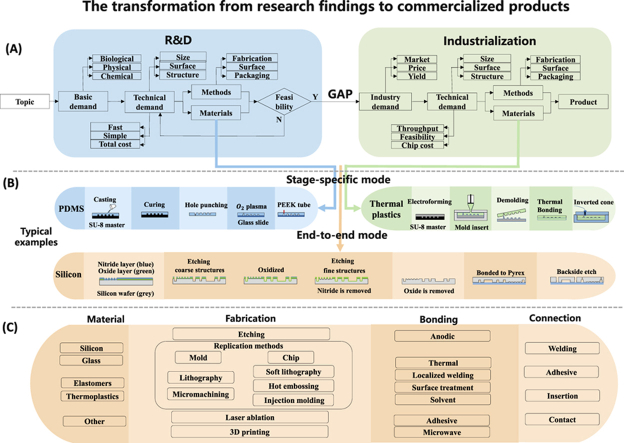

## 1 Introduction

Microfluidics refers to the integration of the basic operating units of sample preparation, reaction, separation, detection, and other essential operating units of the biological, chemical, and medical analysis processes into a micron-scale chip to automatically complete the whole process of analysis. Over recent decades, microfluidics has undergone a significant development. The advancement of miniaturization technology in the 20th century led to the invention of inkjet printers in the 1950s, which enabled precise fluid manipulation. With further progress in processing technologies and the integration of non-conventional materials, a new field called microelectromechanical systems (MEMS) emerged in the 1990s [1,2]. This evolution eventually gave rise to miniaturized total chemical analysis systems (μ-TAS) [3], which are widely regarded as the starting point for microfluidics.

The introduction of polydimethylsiloxane (PDMS) and soft lithography in microchip fabrication by G. Whitesides in 1998 marked a pivotal turning point for microfluidics, leading to explosive development in the field [4]. In 2004, the first study on microfluidic cell culture systems appeared [5], followed by the pioneering work on organ-on-chip technology in 2005 [6]. Polymerase chain reaction (PCR) on chips began commercialization in 2006 [7], while paper-based microfluidics emerged in 2007. By 2008, studies had been conducted using 3D printing to make microfluidic chips, and by 2009, companies and producers had expanded their manufacturing capabilities to include polymers as cost-effective alternative materials to glass. Since then, injection molding has been used for large-scale production of microfluidic chips [8,9].

Over the past 30 years, microfluidics has made significant progress and found applications in a wide range of fields, including biology, chemistry, and information technology [1,[10], [11], [12], [13], [14], [15]]. Some examples of successful microfluidic products include: DNA sequencing products like iSeq, NovaSeq, and Sequel System; PCR products such as the X9 Real-Time PCR System, QX600, and the naica system; point of care testing (POCT) products like GeneXpert and FilmArray; mixers for lipid nanoparticles (LNP) like NanoAssemblr and iLiNP; and organ-on-a-chip devices such as products from Emulate, Hesperos, and TissUse.

Despite numerous publications and quite a few commercialized products in the field of microfluidics, the development of microfluidic-based products has not evolved into a large industry comparable to semiconductor and consumer electronics. One significant challenge is the absence of a comprehensive industrial ecosystem within this field. Unlike the semiconductor industry, where standardized supply chains support large-scale production from initial design to final product, microfluidic industry often sees each product developed independently by different companies. This fragmentation impedes the establishment of a standard supply chain necessary for industrialization and widespread commercialization in microfluidics.

**Fig. 1:**
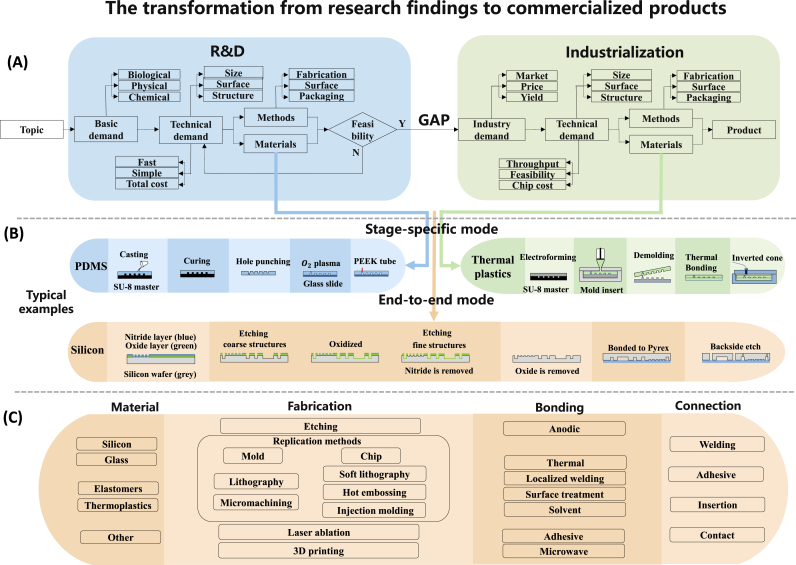
The transformation from research findings to commercialized products. (A) The transformation diagram, starting from the topic selection, R&D, industrialization, and finally, transforming into a product. (B) Typical examples of stage-specific mode and end-to-end mode; i) stage-specific mode: PDMS is used as R&D materials, going through casting, surface treatment bonding and an insertion-based connection [16]; Thermal plastics are used as industrialization materials, going through injection molding, thermal bonding and a contact-based connection. ii) end-to-end mode: silicon is used in both research and industrialization, going through etching and anodic bonding [17]. (C) The common production process of microfluidics. (For interpretation of the references to color in this figure legend, the reader is referred to the Web version of this article.)

The significant gap between research findings and large-scale commercialized products is the main reason for the relatively incomplete industrial chain of microfluidic devices, which has been a significant barrier to the commercialization of many promising research projects. Fig. 1 illustrates the extensive process from initial research findings to commercial products. This lengthy transformation begins with topic selection, research and development (R&D), then industrialization, and finally, the transition of the research project into a commercial product. At the R&D stage, researchers prioritize efficiency and simplicity. However, successful transition from R&D to industrialization requires a shift in focus towards market demand, cost-effectiveness, and production scalability. Fragmentation in demands often leads to differences in materials, production techniques, and other factors, creating gaps that many microfluidic research projects struggle to overcome on their path to commercialization.

The transition to large-scale production of microfluidic devices holds significant promise across various domains. In healthcare, it can provide rapid, cost-effective diagnostic tools for remote or resource-limited areas, enabling early disease detection and personalized medicine. In industry, integrating microfluidics can enhance productivity by automating processes, reducing waste, and improving efficiency, which is particularly beneficial for sectors like pharmaceuticals and food manufacturing. Additionally, the scalability of microfluidic chip production fosters innovation and entrepreneurship, allowing for the development of new applications and products to meet societal and market needs.

This paper provides an overview of the entire process of the transformation from research findings to commercialized products in microfluidic field, discussing the gap between R&D and industrialization, and providing guidelines for bridging the gap. It also highlights the importance of summarizing the different materials used in current products, including silicon, glass, PDMS, and thermoplastics, discussing the properties, fabrication, bonding, and typical products of these materials. Therefore, this paper may offer helpful guidance for developers in the microfluidic field.

## 2 Transformation from research findings to commercialized products

### 2.1 The process of transformation

Overall, the transformation from research findings to commercialized products in microfluidics is a complex and lengthy process that involves various stages, including topic selection, R&D, and industrialization (Fig. 1A).

First, a research topic is selected from a range of fields related to microfluidics, such as physics, chemistry, biology, or medicine. Once the scientific question is identified, researchers assess fundamental material requirements. The basic demands are biological, physical, and chemical properties of materials such as biocompatibility (toxicity to cells), chemical inertness, light transmission, surface properties, and fluorescence background. The properties of materials are shown in Table 1. In the production requirements, the hardness of the material, thermal properties (such as glass transition temperature), and other factors are also considered. Especially for thermoplastic materials, the difference in glass transition temperature greatly affects the production scheme and process. When the same materials are selected in research and industrialization, which can be called end-to-end materials, the same properties can help eliminate the transformation gap. This consistency is crucial, especially for active devices like electrowetting-on-dielectric (EWOD) or next-generation sequencing (NGS), where glass and silicon are used in both research and industrial stages.

**Table 1:** Overview of material properties.

Material	Mechanical property	Thermal property(°C)	Visible range	UV transparency	Biocompatibility	Solvent resistance	Acid/base resistance
Silicon	Rigid	1410	Opaque	none	Good	Excellent	Excellent
Glass	Rigid	500∼821	Excellent	>280 nm	Good	Excellent	Excellent
PDMS	Elastomer	∼80	Excellent	>220 nm	Excellent	Poor	Poor
PS	Rigid	90∼100	Excellent	>300 nm	Excellent	Poor	Good
PC	Rigid	140∼150	Excellent	>360 nm	Excellent	Good	Good
PMMA	Rigid	100∼125	Excellent	>340 nm	Excellent	Good	Good
COC	Rigid	70∼155	Excellent	>360 nm	Excellent	Excellent	Good

In the R&D stage, innovation is essential for creating something new from scratch. This requires frequent experimentation and places great emphasis on efficiency and simplicity—which means being "fast" and "simple". Therefore, it is crucial to pay attention to the overall time from design to chip fabrication, rather than just the cost of the chip itself. Additionally, careful consideration must be given to overall equipment expenses and environmental requirements. Once the R&D requirements are clearly defined, the selection of materials and methodologies—covering fabrication, surface treatment, and packaging—becomes paramount. When choosing the technical demands for R&D, the focus tends to be on selecting the best solution that is "fast" and "simple" within a limited range of material options that fit the size, surface, and structure demands. For instance, PDMS chips manufactured through soft lithography are particularly favored in R&D, especially when cost is not the primary concern, as they perfectly fulfill these requirements.

The industrialization stage of microfluidics is focused on meeting market demand with high yield rates and reasonable costs. The main objectives are to maximize yield rates while keeping costs affordable, and thus need to analyze market demand, yield potential, and average costs of production and equipment carefully. Different fabrication methods such as hot embossing, injection molding, or traditional MEMS technology are suitable for different scales of production to meet market demand. However, the design of the chip plays a critical role in production yield. Factors such as high aspect ratio, smaller draft angle, and inner angles cause difficulties in demolding or manufacturing, leading to yield reduction and additional costs. Therefore, to ensure reasonable costs and competitiveness, complex designs should be avoided. Similar to the R&D stage, the selection of materials, methods (fabrication, surface treatment, packaging), and interfaces undergoes meticulous consideration to optimize the final product.

The development approach for microfluidic products can be categorized into two types based on the materials selected during the R&D and industrialization stages: the stage-specific mode and the end-to-end mode (Fig. 1B). The stage-specific mode employs different materials at each stage to meet distinct requirements. For example, PDMS can be used as R&D material, going through casting, surface treatment bonding and an insertion-based connection [16]; thermoplastics can be used as industrialization material, going through injection molding, thermal bonding and a contact-based connection. A significant challenge in the stage-specific mode is transitioning between two stages, as the materials differ substantially. In contrast, the end-to-end mode maintains material consistency throughout both stages, enhancing the final product's performance while reducing transformation costs. An example of this is the use of silicon in both research and industrialization processes, employing techniques like etching and anodic bonding [17]. However, it's challenging for a single material to satisfy different demands in both R&D stage and industrialization stage, especially when it comes to the product cost and production efficiency. A variety of fabrication processes are available to meet different demands in microfluidics (Fig. 1C). These include soft lithography and surface treatment bonding for PDMS chips, etching methods and anodic bonding for silicon/glass chips, injection molding methods and thermal bonding for thermoplastic material chips. Material and method selection is crucial in both R&D and industrialization, necessitating tailored strategies to meet specific requirements. Specific details about the fabrication process of different materials are discussed in the later sections.

After the lengthy development and transformation process, a wide array of microfluidic products has emerged on the market. For instance, IMEC has developed silicon-based sequencing products like Sequel, while Illumina, the largest sequencing firm, has produced glass-based iSeq and NovaSeq products. Standard BioTools stands out as a successful example in the commercialization of PDMS, employing IFCs as the core. Thermoplastic materials find more widespread use in fields such as digital PCR, digital ELISA, and POCT, with various companies having developed products. Bio-Rad, Stilla, QIAGEN, Thermo Fisher, and Roche have established themselves in the digital PCR market, while Quanterix dominates the digital ELISA market with Simoa. In the POCT field, Cepheid leads with the most products, followed by other companies such as BioFire's FilmArray, etc.

### 2.2 The gap between R&D and industrialization

Despite the significant advancements and rapid growth in the microfluidics field, where numerous research breakthroughs and innovative inventions are continually being made, there remains a considerable gap between academic research and large-scale industrial application. The existing gap hinders microfluidic industry from developing into a large-scale and systematic industry like semiconductor and consumer electronics. Based upon the above discussed process of transformation from research findings to commercialized products, we outline three main aspects regarding the gap between R&D and industrialization.

Firstly, as mentioned above, the R&D and industrialization stages have distinct and stage-specific demands. At R&D stage, researchers focus on developing devices that meet fundamental biological, medical, physical, or chemical requirements with maximum efficiency and simplicity. In contrast, the industrialization stage prioritizes market demand, high yield rates, and cost-effectiveness. This conflict in demands may explain why few products follow end-to-end mode, utilizing the same material throughout both the R&D and industrialization stages. It is often challenging for a single material to satisfy both the efficiency and simplicity required in R&D, as well as the throughput and cost considerations necessary for large-scale production.

Secondly, the transformation process faces several technical and cost-related difficulties. For example, specific design elements in R&D chips such as high aspect ratio, smaller draft angle and inner angles may cause difficulties for large-scale manufacturing with thermoplastics and bring extra costs, though these factors are relatively easy to achieve by materials like PDMS in R&D. To overcome such technical problems, developers often need to modify the original chip design, engaging in what can be considered a "secondary R&D″ stage. On the one hand, the transformation process brings a significant cost in time and money, as well as the cost in communication between laboratories and companies. On the other hand, a great proportion of microfluidic chips are not highly processable due to limitations in their material properties, design complexity, or the constraints of current manufacturing processes.

Thirdly, the absence of a standardized interface within the microfluidic industry has impeded effective communication among different laboratories and companies, resulting in inefficient and ineffective replication. This can be attributed to the fact that the market in the microfluidic field is relatively smaller than that in industries such as mobile phones or automobiles, and more importantly, the demand for microfluidic chips is highly specialized and customized for each sample or case. For microfluidic chips to become commercially viable, they need to be manufactured in large quantities, but there currently lacks application scenarios of such magnitude in the field of microfluidics, which presents a challenge in developing highly reliable manufacturing processes in microfluidics similar to semiconductor industry due to the high cost of equipment and molds.

### 2.3 Guideline for bridging the gap

To effectively bridge the transformation gap from R&D to industrialization in the microfluidics industry, insights can be drawn from the well-established semiconductor industry's supply chain, which typically consists five steps.

1. Material selection: The choice of semiconductor materials, such as Si, GaAs, and SiC, is critical and should strike a balance between performance and cost-effectiveness.
2. Fabless design: Fabless companies like NVIDIA and Apple focus on designing semiconductor chips with flexibility and controllable equipment cost.
3. Fab processing: Foundries, such as TSMC and GlobalFoundries, specialize in converting chip designs into functional semiconductor wafers.
4. Packaging and testing: The manufactured chip will be connected with external pins and packaged into a complete chip module, and passes a series of electrical Functional tests.
5. System integration: Different hardware and software components are finally integrated into a complete electronic system.

For the microfluidic field, standardization across these steps—covering aspects like materials, design, processing, testing, and packaging—is pivotal. Much like the modularity of Lego blocks, the standardized approach in microfluidic industry will facilitate seamless interoperability between different components and stages. For instance, the selection of optimized materials, the design of inner parameters such as aspect ratio, draft angle, and inner angles, the specification of functional components such as pumps, valves, and filters, should all follow a series of industrial standard. It would be highly beneficial if there emerges a “killing application” within the field. Standardization reduces the transformation difficulty and cost, thus paving the way for large-scale production. Once the large-scale production is achieved, the economies of scale will significantly lower overall costs and accelerate both the R&D and commercialization lifecycle.

Practically, both end-to-end and stage-specific modes have offered successful examples of bringing laboratorial microfluidic chips to markets. For the end-to-end modes, Interuniversity Microelectronics Centre (IMEC) is a typical example, which is renowned for developing silicon-based microfluidic chips [18]. Benefited from the innovative and exclusive technology, IMEC has transformed many silicon-based microfluidic designs into commercialized products, like the sequencing product Sequel. However, it should be noted that the cost and pricing of such single microfluidic chip products can be significantly higher than those thermoplastic-based products, which limits their application primarily to high-tech and high-entry-barrier industries.

For stage-specific modes, opting PDMS for R&D and thermoplastics for mass production is a typical pathway, although the gap between these stages is challenging to bridge. Companies like Stratec offer solutions for this approach. Stratec consumerables develops high quality, precise consumables for microfluidic platforms and devices to support a variety of biomedical, diagnostic and laboratory analysis tasks [19]. Similar to semiconductor foundries that handle fab processing, Stratec Consumables provides viable strategies for converting laboratory microfluidic designs into commercial products. Based upon this approach, laboratory researchers can concentrate more on R&D to address specific scientific questions, while the product development and commercialization parts are managed by specialized and efficient team from Stratec consumerables. This transformation strategy offers valuable insights for advancing the microfluidics industry.

## 3 Silicon: typically used in end-to-end mode

Silicon was the first material used for microfluidic chips [20], and is often used in end-to-end developing mode. Silicon-based microfluidics is considered an expensive option, especially for simple passive microfluidic chips comprising basic components such as microchannels and a mixer [17]. However, silicon excels in applications requiring intricate layouts, like microfluidic chips featuring very fine channels, reaction chambers, and sensors. In systems where integration and sensing are the key assets, silicon is the optimal choice.

### 3.1 Material

**Fig. 2:**
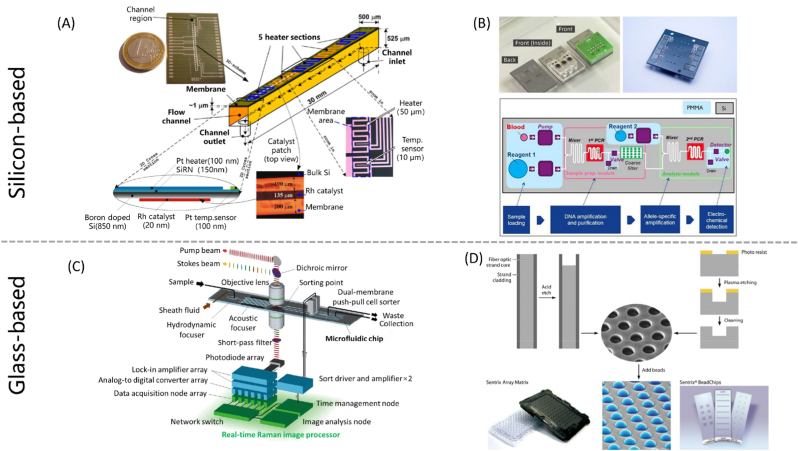
Research findings and commercialized products of end-to-end materials—silicon-based and glass-based microfluidic chips. (A) A microreactor for high-temperature catalytic partial oxidation gas phase reactions made of silicon [24]. (B) PCR chip made by IMEC and Panasonic, based on a silicon-based microfluidic platform [17]. (C) Glass-based microfluidic device used for Raman image-activated cell sorting [35]. (D) Illumina BeadArray chip [36]. (For interpretation of the references to color in this figure legend, the reader is referred to the Web version of this article.)

Silicon is a semiconductor with high elastic modulus, high thermal conductivity, fine biocompatibility, but without transparency to visible light. With high elastic modulus (130–180 GPa), silicon can be fabricated into solid and refined microfluidic chips. As an example, Zhang et al. reported a silicon microfluidic platform that enables monolithic integration of transparent micron-scale microfluidic channels, achieving droplet-assisted electrospray phase separation [21]. The fine biocompatibility of silicon makes it popular in biological applications such as digital polymerase chain reaction (dPCR). The convenient dPCR devices based on silicon microfluidic chips offer a pathway to enable fast and affordable digital microfluidic diagnostic applications [22,23]. Additionally, silicon's ability to form thin films and its high thermal conductivity provide advantages in specialized applications such as high-temperature catalytic microreactors for gas-phase reactions (Fig. 2A) [24]. At present, the most common use of silicon in the microfluidic field is serving as the mold material in soft lithography approaches such as PDMS microfluidics chip fabrication [25]. However, since silicon devices are not transparent to visible light, they're unsuitable for mainstream fluorescence-based detection or direct fluid imaging [26].

### 3.2 Fabrication

Silicon and glass are typical materials for early microfluidic devices, with well-established microfabrication photolithography, etching, and deposition processes [[27], [28], [29], [30]]. Typical silicon patterning techniques are anisotropic wet etching or dry etching by reactive ion etching (RIE) and deep reactive ion etching (DRIE) [31]. As all these processes require high-end equipment and techniques, the cost of silicon and glass microfluidic chips is much higher than polymer ones. Mainly because of the high cost, the wide range of use of silicon in the field of microfluidics only occurred in the early years. However, advancements in microelectronics fabrication have led to substantial cost reductions for silicon chips in these years [21]. Several strategies have been proposed to further mitigate these costs. For instance, Qi et al. developed a strategy to fabricate Si-glass chips with sufficiently low-cost based on reducing the area of each chip and integrating more units into one single silicon wafer [32].

### 3.3 Bonding

Anodic bonding is typically performed between a patterned silicon wafer and a plain glass wafer. In this process, the glass wafer is connected to the negative electrode, while the silicon wafer is connected to the positive electrode. During anodic bonding, high voltage (100 V–1250 V) and high temperature (180 °C-500 °C) are required [33]. Under these conditions, sodium ions in the glass migrate rapidly towards the cathode, resulting in a large electric field at this interface which pulls the two surfaces—silicon and glass—closer [34].

### 3.4 Product

Interuniversity Microelectronics Centre (IMEC) is one of the world's largest research institutes for semiconductor technology, and often in collaboration with other companies to launch silicon-based microfluidic chips [18].

IMEC partnered with Pacific Biosciences to produce the commercial product Sequel System, which utilizes Single Molecule Real-Time (SMRT) technology. This technology is primarily based on Zero-mode waveguides (ZMWs) and innovative nucleotide labeling methods, leading to a higher throughput of data output and exceptional accuracy. Priced competitively at $350,000 comparable to Illumina NextSeq's mid-range devices, the Sequel System boasts the capability to process ten times more human genomes daily, with consumables costing $3000.

IMEC and Genalyte have collaborated to develop disposable silicon photonic biosensor chips that combine IMEC's standard silicon photonic waveguide devices with on-chip grating couplers. This advancement enables high throughput coupling of infrared light with Genalyte's diagnostic equipment. IMEC's silicon photonics platform allows for the miniaturization of complex photonic functions on a single chip and efficient integration of photonics and electronics, thereby reducing manufacturing costs and increasing volume production.

IMEC and Panasonic have jointly developed a product for Single Nucleotide Polymorphism (SNP) detection consisting of a silicon chip with microfluidics [17]. The product consists of a plastic layer with pumps, valves, and a detector, and a PCB to connect the electrical connections of the pump, valves, and detector to the benchtop machine (Fig. 2B). IMEC researchers are currently developing capillary pumps and valves to move fluid through the microfluidic chip without requiring external pumps and valves. They are also introducing a back side metal layer into the platform to make thin-film heaters and temperature sensors, thereby eliminating the external cooling and heating systems.

## 4 Glass: typically used in end-to-end mode

Similar to silicon, glass is another typical material used in end-to-end mode. In the early stages of microfluidics development, glass emerged as a primary material for device fabrication due to its similar processing methods to silicon, beneficial optical properties, and the strength of anodic bonding which allows an excellent resistance to high pressures. These attributes enabled glass to replace silicon in certain applications and fostered its significant advancement in the field.

### 4.1 Materials

Glass offers many advantages in microfluidic applications, including good light transmittance, low fluorescent background, chemical/physical stability, good biocompatibility, relatively low non-specific adsorption, and gas-impermeability. With the characteristic of good light transmittance, glass is widely used for microcells/microparticle visualization as well as optofluidic observation and detection [33,37]. For example, Nitta et al. developed Raman image-activated cell sorting with a glass-based microfluidic device (Fig. 2C) [35]. Due to the electrically insulating and optically transparent properties, glass chips are perfect for microchip electrophoresis (μCE) [38]. With chemical/physical stability, glass is suitable for various chemical analyses, some even under extreme conditions such as high pressure or high temperature. Heiland et al. reported a pressure- and temperature-controllable glass microfluidic chip platform to realize supercritical-fluid chromatography (SFC) [39]. Ultra-thin glass sheet has controllable flexibility, making itself appropriate for constructing microsensors and micro-valves/pumps [33]. Kazoe et al. proposed a femtoliter (fL) volume nanochannel open/close valve fabricated in glass substrates, paving the way for versatile nanofluidic analyses [40]. Glass is also biocompatible, thus suitable for cell culturing and cell analysis, especially serving as the cell culturing plates.

### 4.2 Fabrication

Since glass has a large elastic modulus, the fabrication technique of glass is quite similar to that of silicon. Isotropic wet etching by hydrofluoric acid (HF) is typically employed for glass [31]. However, this method suffers from several drawbacks, such as the high costs per unit substrate area [41], the long processing time, the high environmental requirements, and the difficulty in bonding glass [31]. Therefore, glass microfluidic chips usually cost high compared to polymer ones.

### 4.3 Surface modification

Hydroxylation of glass substrates is easily achieved, and the hydroxyl groups exhibit long-term activity. The hydroxyl group in the channel allows several functionalization methods, including silanization. Silanization of microfluidic substrates enables covalent immobilization of biomolecules onto the microfluidic channels forming a robust and efficient biointerface. Silane serves as a linker between the substrate and the biomolecule, and the termination of the silane needs to be selected depending on the type and conjugation of the biomolecule.

### 4.4 Bonding

There are several types of bonding techniques for fabricating glass-based microfluidic devices. Anodic bonding is typically performed between a patterned silicon wafer and a plain glass wafer. To achieve the bonding of glass-glass wafer at room temperature, surface-activated bonding is required. Without surface activation, two mirror-polished glass substrates sealed by van der Waals forces can withstand only up to 0.6 MPa of shear stress [42]. Surface activation through O2 plasma treatment increases bonding strength to 5 MPa at room temperature [43], while nitrogen microwave radiation can further enhance it to 29.7 MPa [44]. At a low temperature (115 °C), the glass surfaces treated with a calcium solution and 1–2 h of heating could also be bonded together under a high field strength of at least up to 4 kV/cm [45]. Fusion bonding is another common bonding method for glass, which, compared to anodic bonding and surface-activated bonding, has no limitation on the thickness of the glass wafers and no need for a plasma asher/etcher [33]. In fusion bonding, glass substrates are permanently bonded through chemical treatment, low pressure, and annealing [46].

### 4.5 Product

Illumina's early technology was based on the technology of Tufts University professor David Walt, which was based on the etching procedure that resulted in evenly distributed small holes at the ends of the fiber, with highly repeatable and predictable patterns and depths that were orders of magnitude smaller than anything previously reported. During this period, Illumina mainly sold microarray chips (Fig. 2D), of which the SNP chip used similar technology.

In 2007 Illumina acquired Solexa and entered the sequencing market using sequencing by synthesis (SBS) technology. By 2022, more than 90 % of sequencing data all over the world is produced by this instrument [47]. Illumina's sequencing uses a chip called Flowcell, which features billions to tens of billions of nanowells at fixed locations across the surfaces of the patterned flow cells. The structured organization provides even spacing of sequencing clusters to deliver significant advantages over non-patterned cluster generation. Illumina's products on the market include a compact benchtop system (iSeq sequencing system, $20,000) for rapid analysis of small samples, and the high-end NovaSeq ($1 million) for large-scale sequencing projects.

## 5 PDMS: typically used in R&D stage

Since its introduction as a microfluidic substrate in the late 1990s [3], PDMS has catalyzed explosive development in the R&D of microfluidics. Due to the simple and convenient moldability of PDMS, it is widely used in the R&D stage. PDMS is also employed in some commercialized products, such as the ones developed by Standard BioTools.

### 5.1 Materials

PDMS possesses many beneficial properties as a microfluidic material, such as high optical transparency, low autofluorescence, good biocompatibility, low toxicity, easy mouldability, low elastic modulus, and gas permeability [[48], [49], [50], [51]]. The soft nature of PDMS allows for many interesting applications, such as the famous Quake valve [52]. In R&D, PDMS has a wide range of applications in different fields, such as micropumps [53], microvalves [54], catheter surfaces [55], dressings and bandages [56], optical systems [57,58], vitro study of diseases[59,60], and implantation [61,62].

### 5.2 Fabrication

The most common method to fabricate PDMS is soft lithography [63]. Soft lithography methods have no limitation in precision, nano/micro structure microfluidic chips can all be fabricated as long as the mold itself has the precision [64]. Conventional photolithography, where the negative photoresist of SU-8 is photopatterned on a silicon wafer to create the protrusions of a microchannel network [48,65], is commonly used to fabricate the mold. This approach allows laboratories to produce their own Lab-on-a-Chip (LOC) devices. Importantly, multiple layers can be stacked to create complex fluidic designs [66], such as the Quake valve [52] and 3D microfluidic devices [[67], [68], [69]]. However, the necessary curing time (1–2 h) significantly extends the production cycle of PDMS [70].

### 5.3 Surface treatment

Surface treatment is commonly employed to modify surface properties of PDMS. Plasma treatment is the most commonly used surface modification method for PDMS [71]. Oxygen plasma treatment can produce hydrophilic functional groups on the surface of PDMS, but because of the migration of oligomers from the interior of PDMS to the surface, hydrophobic reversion occurs easily. Another method is UV treatment, which is nearly an order of magnitude slower than plasma treatment. However, UV treatment allows for much deeper modification of the PDMS surface without inducing cracking or mechanical weakening of the PDMS, and is much safer. Chemical vapor deposition (CVD) has been used to fabricate PEO-functionalized microchannels to resist fibrinogen adsorption [72]. This technique produces thin, coherent layers that adhere well to various substrate films, and may be prepared from monomers, which cannot be polymerized by conventional means. Sol-gel chemistries are also widely used as thin-film coatings due to their low reaction temperature and easy control of porosity to allow ion transport. The glass coating on the electrodes and PDMS surface improves the bonding strength, chemical resistance, and durability for reuse [73].

### 5.4 Bonding

Surface treatment is widely used to increase the surface energy of polymers [74]. Oxygen or air plasma activation is the most common way to bond PDMS layers [[75], [76], [77], [78], [79], [80]]. After the activation, a heating process is usually needed to promote bonding strength [[81], [82], [83]]. Ultraviolet/ozone (UVO) is another method to activate the surface [84]. Compared to the plasma activation process, UVO has the benefit of performing in the air without requiring a vacuuming system. Although the surface treatment process is easy to operate, the main drawback is that the bonding is irreversible once the layers are put together, which restricts adjustments. In multilayer soft lithography, “off-ratio bonding” is commonly used [85]. This technique takes advantage of the fact that PDMS is a two-component elastomer consisting of a base, also known as potting compound, and a crosslinking agent [85].

### 5.5 Product

**Fig. 3:**
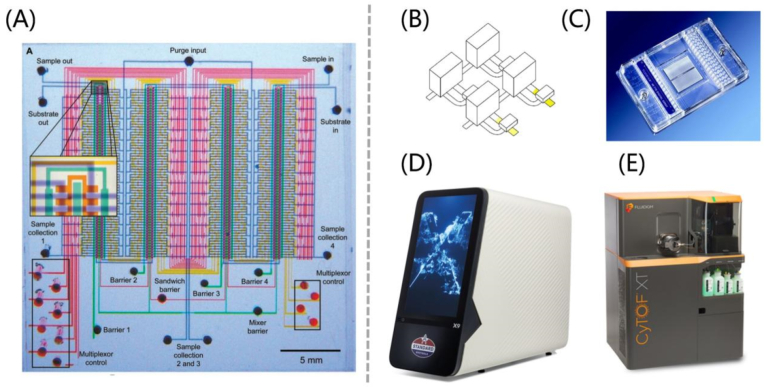
Research findings and commercialized products of end-to-end materials—PDMS-based microfluidic chips. (A) Optical micrograph of the microfluidic comparator chip using the Quake valve [86]. (B) Schematic of Standard BioTools' core technique—integrated fluidic circuits (IFCs) (C) A digital array IFC chip [87]. Standard BioTools' two most important core technologies [88]: (D) the X9 one-stop microfluidic gene analysis system and (E) mass spectrometry flow cytometry (CyTOF).

Standard BioTools, formerly known as Fluidigm, is based on the core technology—Quake valve (Fig. 3A), invented by Professor Stephen Quake of California Institute of Technology in 1998. Now Standard BioTools has made it into a series of chips with different functions, with core components named Integrated fluidic circuits (IFCs) (Fig. 3B). Standard BioTools has developed two most important core technologies, a new generation of medium and high-throughput genomics platforms—the X9 one-stop microfluidic gene analysis system (Fig. 3C), as well as mass spectrometry flow cytometry (CyTOF) and imaging mass spectrometry flow cytometry (IMC) (Fig. 3D and E). However, the relatively high cost of PDMS results in the elevated pricing of their core accessory IFCs, and each chip can be sold for up to several hundred dollars. Therefore, Standard BioTools’ products are still more popular in basic research. To occupy a larger share in clinical applications, further improvements and breakthroughs are essential, which is also a challenge for large-scale production with PDMS.

## 6 Thermoplastics: typically used in industrialization stage

Thermoplastic polymers are most commonly used through the hot embossing and injection molding process because of their cost-effectiveness, low molding temperature requirement, light weight, and other different properties [89]. Therefore, they are widely used in the industrialization stage. Currently, a considerable portion of products applied in the market were developed using materials like silicon, glass, and PDMS in the early stages, and then replaced with thermoplastics in large-scale production.

### 6.1 Materials

Thermoplastics are a class of synthetic polymers that are moldable when heated to their glass transition temperature and retain their shape when cooled. Thermoplastics are easy to recycle and do not exhibit any chemical changes when heated or cooled multiple times. Among them, the most used thermoplastic materials are Polystyrene (PS), Poly (methyl methacrylate) (PMMA), Polycarbonate (PC), and Cyclic-olefin copolymer (COC).

#### 6.1.1 PS

PS is optically transparent, biocompatible, inert, and rigid. Its surface can be easily functionalized and its naturally hydrophobic properties can also be modified [90]. PS is a commonly used material in cell culture [91], outperforming PDMS in certain aspects of cell biology applications. PS can be easily converted from hydrophobic to hydrophilic by plasma treatment and can stay hydrophilic for 4 weeks, which is approximately four times longer than PDMS [92]. Minhwan Chung et al. created a tumor sphere-induced angiogenesis model for drug screening utilizing an injection-molded plastic array 3D sphere culture platform made of PS [93]. And Ki-Young Song et al. created a PDMS-PS microfluidic cell culture system in which primary skeletal muscle progenitor (SMP) were differentiated and matured [94].

#### 6.1.2 PMMA

PMMA, the least hydrophobic polymer in common plastics, is one of the most commonly used materials in microfluidic systems [95]. PMMA is relatively inexpensive, has an elastic modulus of 3.3 GPa, and has good optical clarity from the visible into the UV [96]. It exhibits biological compatibility, gas impermeability, and ease of micromachining at relatively low temperatures. In comparison to PMDS, PMMA's low air permeability limits its use in biological culture. PMMA can be combined with electrophoresis and is easy to manufacture and modify. PMMA also has environmentally friendly properties because it can be broken down into MMA at high temperatures and recycled.

#### 6.1.3 PC

PC exhibits optical transparency, biocompatibility, inertness, high impact resistance, low moisture absorption, good processing performance, and exceptionally high softening temperature, making it desirable for DNA thermal cycling applications [97]. While similar to PMMA in some respects, PC is more expensive, stronger, and suitable for a wider temperature range. However, PC is readily attacked by diluted alkalis, aromatic, and halogenated hydrocarbons. PC is also a commonly used material in biological and medical analysis, including PCR [98].

#### 6.1.4 COC

COC has very good optical properties [99], biocompatibility as well as high chemical resistance [100,101], low moisture absorption, high water resistance, high heat resistance, and high dimensional stability. Being a relatively new polymer type, there are several commercial COCs derived from various cyclic monomers and polymerization methods. Transparency to ultraviolet light makes it an effective material for integrated circuits in bioassays. However, COC also has drawbacks such as relatively brittleness and low thermal diffusivity. Because of the hydrophobicity, when COCs are exposed to biological tissues or liquids, they are susceptible to spontaneous nonspecific protein adsorption and cell adhesion, which makes them not the best choice for research involving drugs.

#### 6.1.5 Other polymers

Many other polymers are used to make microfluidic systems, including polyethylene (glycol) diacrylate (PEGDA), thermoplastic polyurethanes (TPUs), polyetheretherketone (PEEK), polyethylene terephthalate (PET), polyvinyl chloride (PVC), Teflon, etc.

### 6.2 Fabrication

Replication methods are great cost-saving for mass production if continuous copying of the mold can be achieved at micro- or nanometer scales [31]. The accuracy and properties of these methods heavily rely on the quality of the master.

Different methods are used to fabricate the master, with conventional photolithography being the most commonly used method. However, additional processes such as electroplating are typically required. Nickel and its alloys or copper are commonly used as electroplating materials. Another widely adopted method is micromachining [82,[102], [103], [104], [105], [106], [107], [108], [109], [110]], which excels at fabricating high aspect ratios with inclined angles on side walls, thus beneficial for demolding [111,112]. Micromachining is extensively used in industry to create low-cost molds and enables the manufacture of stainless steel materials. Compared to alternative methods, micromachining has limitations in achieving very fine feature sizes and controlling surface roughness.

Replication procedures are usually used in large-scale production, because of their affordability and adaptability to various materials and product designs. In the hot embossing process, the microchannel can achieve replication speeds of 10–30 min per cycle depending on the heating and cooling conditions; by contrast, injection molding and continuous roll-to-roll imprinting methods can finish a replication cycle in a matter of seconds.

#### 6.2.1 Hot embossing

**Fig. 4:**
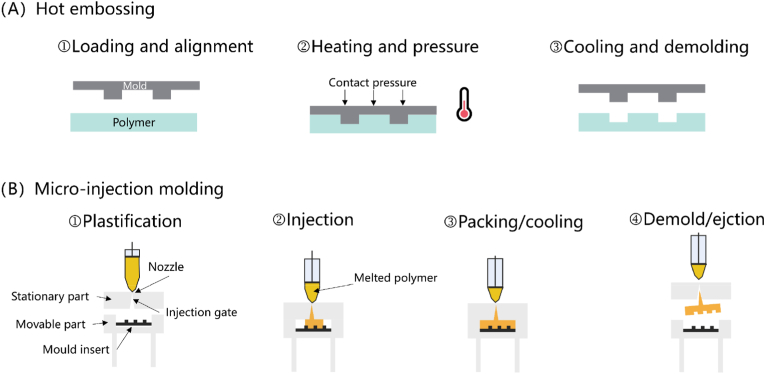
Schematic for the common large-scale fabrication methods [31]—(A) hot embossing and (B) injection molding.

Hot embossing, which is to replicate micron-scale structures by application of pressure and temperature (Fig. 4A) [[113], [114], [115], [116]], has been widely used in the past 10 years [117]. Hot embossing can also be divided into different categories such as traditional plate-to-plate hot embossing and roll-to-roll hot embossing [118,119]. At present, EDEN TECH has implemented a method where silicon molds are replicated using epoxy resin, followed by rapid chip production using the Sublym hot embossing machine, further reducing the cost and requirements of the hot embossing process. The problems with hot embossing include how to fill the material into the mold during the heating process to achieve maximum accuracy and how to demold the chip without affecting the microstructure [120].

Compared to injection molding, hot embossing has lower equipment costs and better replication quality [[121], [122], [123], [124], [125], [126], [127]]. As the temperature in hot embossing is lower, low residual stresses and less shrinkage are observed in the embossed part, which makes its equipment cheaper [122]. However, hot embossing has longer cycle times, typically taking minutes per cycle, which limits its throughput compared to injection molding, and makes it particularly suitable for medium-scale production (∼100–1000/month).

#### 6.2.2 Injection molding

For mass production, injection molding is highly preferred especially for large quantities, due to its low cost, short cycle times, ability to create versatile shapes, simple automation, and simultaneous shaping of bulk and surface structures [[128], [129], [130]]. Micro injection molding follows steps similar to those of conventional injection molding (Fig. 4B) [113], but polymer flow behaviors differ significantly at the microscale compared to the macroscale [131,132], making filling more complex [133]. Polymers with proper flow properties and low viscosity at high-temperature are required [134], including PS [135], COC [134,[136], [137], [138]], PMMA [137,139], PC [140,141], PEEK [142,143], etc. [[144], [145], [146], [147], [148], [149]].

The injection molding process is complicated, influenced by numerous parameters [150]. Increasing mold temperature is considered the most useful way to improve quality [129,135,[151], [152], [153], [154], [155]]. Demolding conditions also affect the result [136,156,157], and there are two different main demolding methods. The first uses demolding chemical surface agents [136,[158], [159], [160], [161]]; the second is with a mechanical ejector such as pins, blades, rings, sleeves, and stripper blades [151,156,162].

Compared to hot embossing, despite the high equipment price and complicated factors that will influence the results, injection molding offers mass-scale potential due to its replicability and cost-effectiveness [163]. Moreover, because of the capability to use a wide range of materials and fully automated processes that reduce cycle times, hot embossing is highly appealing for industrial applications [31,164]. Nevertheless, both hot embossing and injection molding face challenges in fabricating high aspect ratio features, particularly those without draft angles typically made by lithography processes [165]. For injection molding, the problem is more severe due to higher thermal stresses. Therefore, the designs must take into account many factors, from polymer selection and mold conditions to processing parameters and the part design [166].

### 6.3 Bonding

**Fig. 5:**
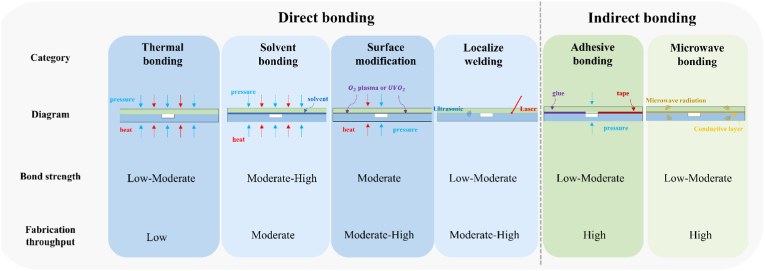
Summarization of the bonding methods for the common large-scale production materials—thermal plastics [167].

The bonding step is crucial in the fabrication of microfluidic chips as it directly impacts the maximum pressure the chip can withstand, thereby ensuring the availability of the channels. The common bonding method, bonding strength, and throughput of thermal plastics are summarized in Fig. 5.

#### 6.3.1 Thermal bonding

Thermal bonding is to heat the thermoplastics near or above their glass transition temperature (Tg) and simultaneously apply pressure to achieve bonding [168,169]. It is suitable for most thermoplastic materials, including PC [170], PMMA [[171], [172], [173]], COC [174], etc. For thermal bonding, the heating temperature will have a greater impact on the structure and affect the accuracy of the structure. In industrial settings, programmable machines or high-throughput roller presses are commonly used for thermal bonding [175]. A major challenge of thermal bonding lies in adjusting bonding conditions for different substrate materials to achieve optimal bonding strength and preserve channel integrity. Moreover, the entire bonding process typically occurs within a clean room environment.

#### 6.3.2 Solvent bonding

Solvent bonding is widely used because of the advantage of low cost, good optical clarity, fast bonding process, low-temperature requirement, and high bond strength [167]. In solvated polymers, chains intertwine extensively between surfaces, resulting in exceptionally strong bonds. Methods such as pipetting [176], spray coating [177,178], and soaking [179] are often used to uniformly spread the solvent. Plasma treatment [180] and UV irradiation [177,181] are also used to improve the bonding quality. Besides, vapor solvent evaporation is also extensively used for uniform solvent deposition, resulting in a clogging- and distortion-free microchannel [181]. The biggest challenges for solvent bonding are the clogging and distortion of the channels. Another major challenge is the poor bonding coverage near the free edges [182].

#### 6.3.3 Localized welding

The method of local welding is to use some devices to concentrate the energy on some positions for targeted bonding, or to heat the whole chip to achieve the purpose of bonding [169]. Both ultrasonic [[183], [184], [185], [186], [187]] and laser welding [[188], [189], [190], [191]] are typical weld-bonding processes [192]. Ultrasonic bonding uses the propagation of ultrasonic sound at 20–40 kHz or higher frequencies to create local melting between a sonotrode and an anvil [193]. It offers rapid bonding at low temperatures with high bond strength. However, ultrasonic bonding can lead to polymer shrinkage and uneven energy distribution. Laser welding is another localized welding method [[188], [189], [190], [191]], requiring specific conditions that optical transparency in the upper plastic layer and absorbency in the lower layer to create melting [194]. The bonding strength can be as high as 6 MPa [194].

#### 6.3.4 Surface treatment and modification

The surface modification techniques of thermoplastic materials are similar to that of PDMS. Physical surface modification methods mainly include plasma treatment and UV surface treatment. Plasma treatment has been used to improve the hydrophilicity of COC [195], PMMA, and PEEK [196], while sometimes reducing the hydrophilicity of thermoplastics using oxygen with CYTOP-polyaniline or HFTTCS [197,198]. UV treatment has been used to create biofunctionalized surfaces on COC [199] and PS [200], capable of imparting a gloss appearance to the polymer surface. As for chemical surface modification, it uses chemical reagents to increase the surface energy or enhance bonding strength of the microfluidic device. The chemical reagents used include (3-aminopropyl)triethoxysilane (APTES) [201], (3-triethoxysilyl)propylsuccinic anhydride (TESPSA), 3-(trimethoxysilyl)propyl methacrylate (TMSPMA) [202], etc.

#### 6.3.5 Adhesive bonding

Adhesive bonding is widely adopted in conventional plastic processing. Both liquid-form adhesive and dry adhesive are used for thermoplastic bonding. Adhesive bonding remains favored in mass production due to its rapid, simple and cost-effective process [169]. Bonding with adhesives is simple and convenient, though one of its challenges, similar to solvent bonding, is the potential for channel clogging. The liquid-form adhesive can be UV curable adhesive [203,204], epoxy adhesive [[205], [206], [207]], optically clear adhesive [208], PMMA solution [209], hot-melt adhesive [210], wax [211], etc. [209,212,213] To avoid clogging issues, different methods, such as spin coating [204,208,209,214,215], direct adhesive printing [216], contact printing method [216], capillarity-driven adhesive delivery [205] and selective stamp bonding [206,207] have been introduced for the application of adhesive on bonding surfaces. Besides liquid-form adhesive, dry adhesive films are also used. The dry adhesive includes pressure-sensitive adhesive, adhesive film, or adhesive tape, such as commercialized pressure-sensitive adhesive (PSA) tapes [207,[217], [218], [219], [220]]. Dry adhesive films are preferred over liquid-form adhesive by companies because of the less clogging problem, more reliability, simple process, and less cost.

#### 6.3.6 Microwave bonding

Microwave bonding technique uses microwave to heat the conductive layer in the bonding interface to achieve bonding between thermoplastic substrates [221]. Compared to other methods, the clogging problem happens less in microwave bonding. To improve the quality or simplify the process, different methods have been proposed to improve the conductive layers, such as a conductive polymer (Polyaniline) [222] which can be introduced through screen printing [223] or interfacial capillary force [224], as well as the use of gold/chromium layer [221,225]. One advantage of microwave bonding is its capability for selective heating and localized melting, which requires only a low-cost facility. However, the addition of conductive materials can increase both the complexity and the budget of the bonding process.

### 6.4 Product

This part introduced provides a brief overview of the several most renowned and classic microfluidic products, which opt silicon/glass/PDMS/optical fibers for R&D and thermoplastics for production.

#### 6.4.1 Point of care testing (POCT)

PCR was a key application in the microfluidics field, with discussions about the development of PCR-Microreactors dating back as early as 1994 [228]. As the field progressed, PCR found its way into various microfluidics applications, including the POCT field. POCT involves clinical testing conducted right next to the patient, typically without the need for a clinical examiner. It is a novel technique that enables immediate analysis at the sampling site, eliminating the need for complicated specimen handling procedures in laboratory testing, and providing rapid test results. Microfluidics' high throughput, low cost, and miniaturization advantages make it an ideal fit for POCT.

**Fig. 6:**
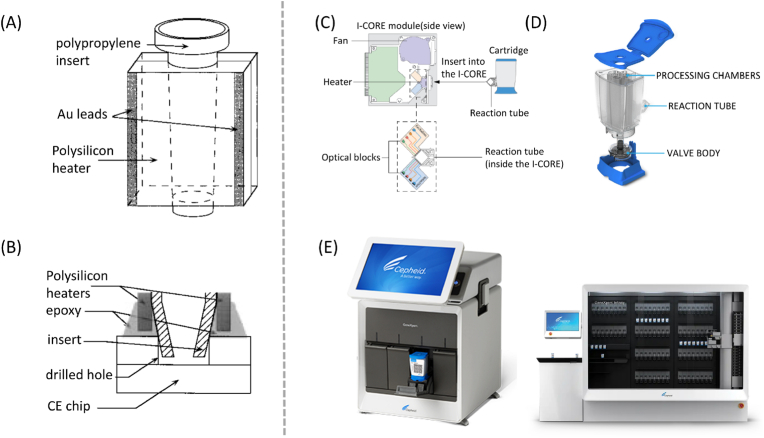
Point of Care Testing: (A) Expanded view of the micro-fabricated PCR chamber [226]. (B) Expanded cross-sectional view of the junction between the PCR and CE devices [226]. Cepheid's core technic [227]: (C) I-CORE module, (D)cartridge and (E)the GeneXpert product.

Cepheid, a dominant player in the POCT field, initiated its development by commercializing Lawrence Livermore National Laboratory's high-speed nucleic acid amplification technology to establish a foothold in the PCR market. In 1995, M. Allen Northrup et al. successfully created a Microfabricated DNA Analysis Device integrating a Lawrence Livermore National Laboratory's silicon-based PCR device (Fig. 6A) and a capillary electrophoresis (CE) device made of glass (Fig. 6B) [226]. Subsequently, cheaper plastics were gradually employed in product manufacturing. Presently, Cepheid's GeneXpert products (Fig. 6E) represent the world's most sophisticated automated molecular diagnostic platform, integrating sample preparation, nucleic acid amplification, and detection into a kit. The product primarily comprises an I-CORE (Fig. 6C) module and chip (Fig. 6D), featuring a lid, a reagent pool, a base, and a reaction tube. While materials such as PDMS and PE can be used for the crucial reaction tube part, commercial usage still relies on PP.

#### 6.4.2 Digital ELISA

**Fig. 7:**
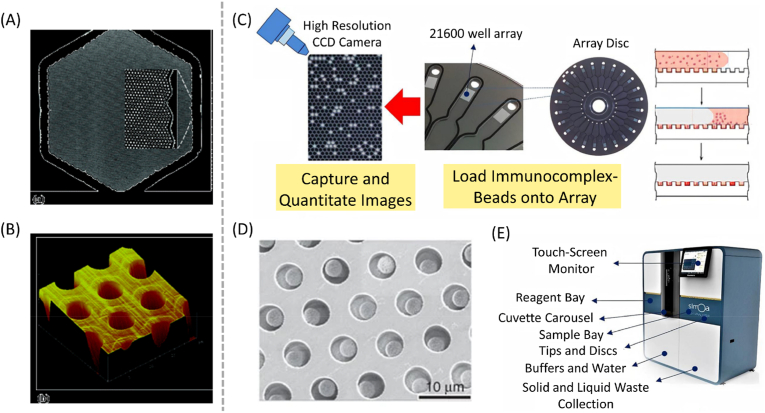
Digital ELISA: (A) Entire fiber array and close-up microscope images of the fiber bundle, emphasizing the regularity of both the array and each individual optical fiber [229]. (B) AFM image of a portion of the etched surface, showing wells created from the etching process [229]. (C) Schematic illustration of the work flow of the SiMoA Digital ELISA [230]. (D) The scanning electron micrograph of a small section of a femtoliter-volume well array after bead loading [231]. (E) Quanterix's digital ELISA product [230].

Digital ELISA is a sensitive and quantitative immunoassay method used to detect and measure proteins or other biomolecules in biological samples. It is similar to digital PCR in its underlying concept and has the ability to detect even low concentrations of proteins or biomolecules. The idea for digital ELISA was born out of David Walt's research at Tufts University, where he was working on developing a digital assay that could detect individual molecules. David Walt was the founder of Quanterix and the scientific founder of Illumina. Digital ELISA chip and the previous Illumina microarray chip have similar requirements, and the development of both started with the regular pores created by etching. Walt believed that if he could develop a sensitive enough assay, it would revolutionize the field of diagnostics and lead to earlier disease detection, better treatment outcomes, and improved patient care. As early as 2006, Walt published a paper about using femtoliter arrays for digital concentration readout of single enzyme molecules [229]. These arrays utilized optical fiber bundles containing approximately 2.4 × 10^5^ individual 4.5 μm diameter optical fibers as substrates for femtoliter reaction vessel arrays. Fig. 7A and **B** illustrate the surfaces of fiber optic bundles and etched wells [229]. In 2007, David Walt led a technical team from Harvard University to establish Quanterix, and then carried out research on single-molecule detection technology for 3 years. Finally, in 2010, David Walt and Duffy published a paper on SiMoA (Single Molecule Array) technology (Fig. 7D) in Nature Biotechnology, which became the core patent of Quanterix [231]. Later, Quanterix officially launched their product HD-1 (Fig. 7E) [230]. SiMoAs make use of arrays of femtoliter-sized reaction chambers that can isolate and detect single enzyme molecules (Fig. 7C) [230]. The limit of detection (LOD) of SiMoA can be 2–3 orders of magnitude higher than the traditional ELISA method, reaching the femtogram level (fg/ml). It is based on the use of microbeads that are coated with specific antibodies and then mixed with a biological sample. The microbeads are then read by an instrument that can detect and count individual molecules.

#### 6.4.3 Digital PCR

Digital PCR (dPCR), first proposed by Vogelstein and Kinzler in 1999 [234], is a nucleic acid quantitative analysis technology rapidly developed in these years. Unlike traditional qPCR, which relies on amplification curves and Ct values to infer target molecule concentrations, dPCR directly counts nucleic acid molecules present in a sample. This absolute quantification is achieved through partitioning the sample molecules into thousands to millions of individual reactions, each containing a single molecule or a few molecules. There are two main types of common dPCR on the market: droplet dPCR (ddPCR) and chip dPCR (cdPCR) technology. The cdPCR is mainly represented by Standard BioTools introduced before. Due to the high cost of cdPCR to manufacture chips, ddPCR is becoming more and more welcomed by enterprises.

**Fig. 8:**
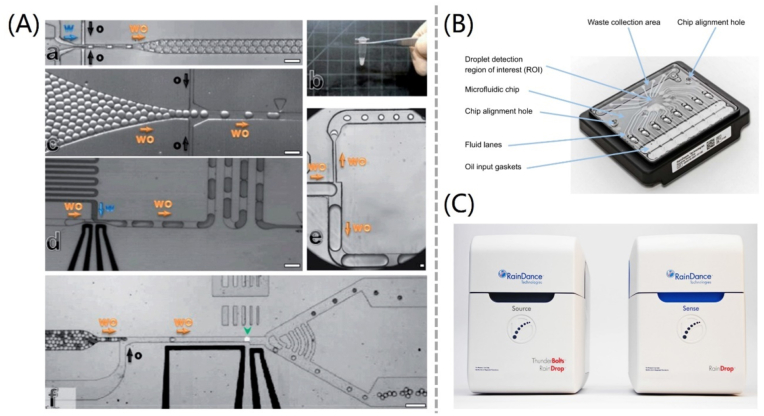
Droplet digital PCR: (A) Manipulations with droplet microfluidics [232]. (a) A droplet-producing device. (b) One million droplets stored in a 200 μL microtube. (c) A reinjection device. (d) A picoinjection device. (e) A splitting device. (f) A sorting device. Rain Dance's (B) chip structure and (C) product [233].

Significant advancements in dPCR have been facilitated by the integration of microfluidic technologies, with PDMS as a key material. Professor David Weitz, a co-founder of Raindance Technologies along with Jonathan Rothberg in 2004, is a pioneer in ddPCR through extensive research in droplet microfluidics. Raindance's RainDrop Digital PCR technology works by partitioning a sample into millions of droplets, each containing a single DNA or RNA molecule and the necessary reagents for PCR amplification. This approach enables highly parallel, high-throughput analysis of multiple samples in a single run, with precise control over reaction conditions and minimal sample loss. Fig. 8A shows the research on droplet microfluidics using PDMS as the development material [232], as well as the Raindance chip (Fig. 8B) and product (Fig. 8C) that uses droplet microfluidics.

**Fig. 9:**
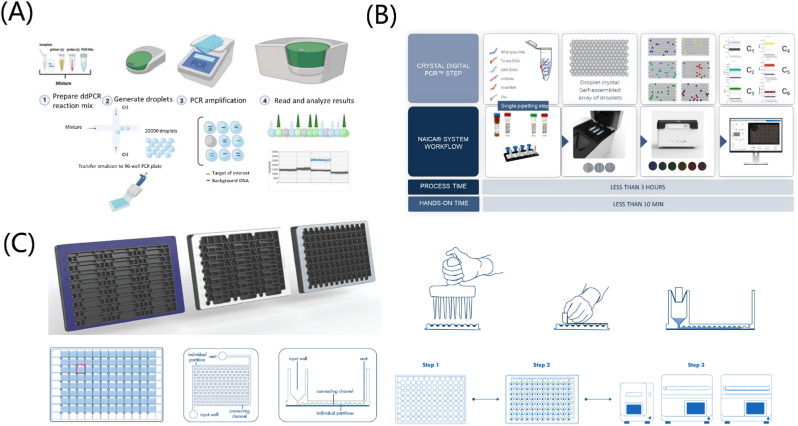
Other digital PCR products: (A) The most famous droplet dPCR (ddPCR) company—Bio-Rad's QX product and its principle and chip structure [235]. (B) Stilla's naica system's workflow and the crystal digital PCR steps [236]. (C) The structure of QIAGEN's QIAcuity's chip structure and the workflow [237].

Currently, Bio-Rad holds the largest market share in the use of ddPCR, after it acquired Raindance in 2017. In 2022, Bio-Rad launched the latest edition of their ddPCR system, the QX600 microdroplet digital PCR system. This new system offers six-color multiplex technology and retails for around $ 260,000 (Fig. 9A) [235].

Several companies offer dPCR products based on different solutions, such as Stilla's naica system, which uses hybrid chips, and QIAGEN's QIAcuity, which is based on the physical segmentation of chips. Stilla's naica system utilizes crystal digital PCR and provides Sapphire and Opal chips (Fig. 9B). The sample is divided into a large array of thousands of individual droplet crystals, and each droplet crystal is labeled with fluorophores before amplifying the nucleic acid molecules, which can be read using up to six different fluorescence channels. QIAcuity's innovative nanomicroplate employs microfluidic technology, and after configuring the PCR reaction system, the instrument automatically dispenses the sample into the nanomicrowell of the microplate and seals each well (Fig. 9C). The nanomicroplate technology can achieve physical separation, ensuring uniform droplet size dispensed into each nanowell, with no droplet rupture, fusion, or cross-contamination.

## 7 Other fabrication methods

In addition to the commonly used fabrication methods introduced before, there are also other emerging methods in the field of microfluidics, such as laser ablation and 3D printing. Like the soft lithography method used for fabricating PDMS, both laser ablation and 3D printing are considered fast, simple, and cost-effective approaches, making them particularly suitable for R&D stage. It is worth noting that for 3D printing, despite not being widely adopted in the microfluidic industry, its distinctive capabilities for constructing three-dimensional structures, combined with the significant advancements in recent years, present it as a potential alternative method for manufacturing microfluidic devices.

### 7.1 Laser ablation

The principle of laser ablation is to use a high-intensity laser to focus on the location we need to remove, and the energy of the beam is concentrated at that point to evaporate the material [238]. The laser sources used in laser ablation can be classified based on their wavelengths (UV/excimer lasers and infrared lasers) or the time scale of their pulse durations (millisecond, microsecond, nanosecond, picosecond, and femtosecond lasers [239]). At present, many materials can be processed by laser ablation, most of which are polymers such as PMMA [240], COC [241,242], PS, PC, PET, and PDMS, but sometimes glass and other materials can also be processed.

For laser ablation, the drawback includes non-uniform cavity depth and slanted sidewalls [31,243]. The depth of single irradiation is typically around 1 μm, thus deeper depth requires multiple shots, resulting in a relatively rough channel surface [244,245]. Additionally, effective methods are necessary for removing residual material [246]. Despite these challenges, laser ablation has several advantages, such as improved accuracy, shorter processing time, no pollution in the processing process, and wide acceptance of workpiece materials [247].

### 7.2 3D printing

**Fig. 10:**
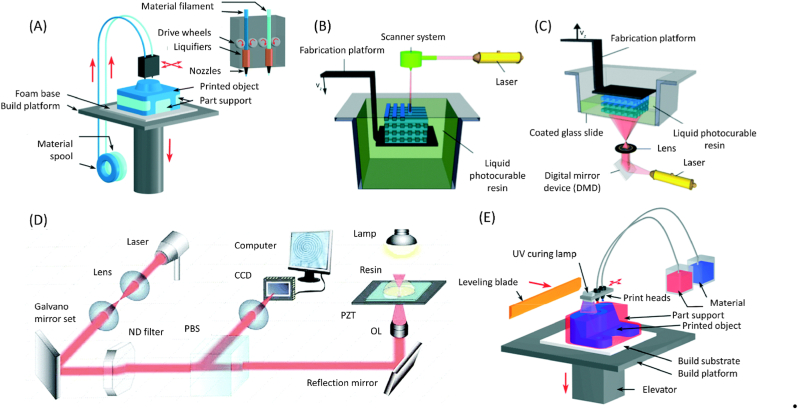
3D printing, the emerging method that is still more applied in R&D [255]. (A) fused deposition modeling (FDM). (B) Stereolithography (SLA). (C) digital light processing (DLP). (D) two-photon polymerization (2PP). (E) multi-jet modeling (MJM).

3D printing, or additive manufacturing, generally constructs three-dimensional objects through computer-aided design (CAD) and builds them layer by layer [248].This technology has been widely used in the fabrication of microfluidic chips, due to its ability to realize complex 3D structures instead of 2.5D structures without the complicated overlay and precise alignment [249]. he main methods of 3D printing include fused deposition modeling (FDM), stereolithography with digital light processing (DLP), two-photon polymerization (2PP), and multi-jet modeling (MJM)(Fig. 10).

FDM is the lowest-cost 3D printing technology, with a machine cost of as low as $300–2000, and a material price of about $20/kg [250,251]. In FDM, filaments are heated and extruded through a nozzle to create a 2D plane, followed by layer-by-layer construction of a 3D object [252]. The materials used include a variety of thermoplastic filaments that are close to those in hot embossing, which may facilitate the transition from prototyping to mass production [249].

Stereolithography with digital light processing (DLP) has been commercially applied, with a machine cost of $2000–5000 [251,253]. It achieves a resolution of approximately 30 μm and a material price of about $200–400/kg, but with limited biocompatibility [254]. Stereolithography uses ultraviolet light to illuminate specific areas of the surface of a liquid photosensitive resin, which cures to form one layer of the object [255]. Method for controlling the illumination area includes scanning and digital light processing (DLP). The main limitation of this technology is the need for a fast-curing and biocompatible photosensitive resin.

Two-photon polymerization (2PP) technology has the highest resolution but with equipment costs of up to millions of dollars. 2PP technology is based on the same light curing principle as stereolithography, utilizing a near-infrared femtosecond laser to initiate photochemical reactions precisely at the focal point. This process solidifies voxels within a liquid material, enabling the construction of intricate three-dimensional structures with resolutions reaching tens of nanometers [256].

Multi-jet modelling (MJM) has been adopted by commercial companies with machine costs from $80,000 to more than $100,000 [251]. Its printing accuracy is close to DLP, with the best resolution at about 15 μm [257]. In MJM, after applying the photopolymer to inkjet 3D printing (i3DP), droplets of both building and supporting materials are deposited from multiple inkjet printheads and cured layer by layer using light [258]. Because multiple chips can be printed at the same time, MJM has a relatively low time cost to manufacture chips. However, the lengthy post-processing to remove the support material limits its efficacy in rapid prototyping [258].

## 8 Conclusions

Microfluidics has made significant progress in various fields over the past 30 years. As MarketsandMarkets predicts, the global microfluidics market in terms of revenue was estimated to be worth $22.3 billion in 2023, and will grow at a compound annual growth rate of 13.0 % from 2023 to 2028 [259]. However, the industry is still considered nascent and lacking complete development. Despite current commercialization in various areas, there still exist gaps that need to be filled for a complete transition from academia to industry. This paper discusses the transformation gap between research advancements and commercial products, while providing an overview of the materials currently used in microfluidic devices, including their fabrication methods and typical applications. Our aim is to offer insights that can help developers better understand the challenges facing the microfluidic field and provide useful guidance for overcoming these obstacles.

The relationship between microfluidics and productivity liberation is paramount. The large-scale production of microfluidic technology holds significant potential for enhancing productivity across various industries. By improving production efficiency, reducing costs, and optimizing resource utilization, mass production of microfluidic chips can yield substantial benefits. Its precise fluid control and automation capabilities streamline complex processes, minimize human intervention and enhance throughput and operational efficiency on production lines. Additionally, the application of microfluidic technology enables higher precision and stability in production, leading to improved product quality and reduced waste, further amplifying productivity. However, it should be noticed that microfluidic devices usually have poor production rate at the milliliter scale due to the inherent characteristics of microfluidics, which limits their applicability in processes requiring larger-scale production volumes.

In conclusion, although there are challenges that impede the transition of microfluidic devices from research prototypes to large-scale commercial products, the ongoing development and application of microfluidic chips will ultimately lead to the integration of this technology into everyday life. As this field advances, microfluidics will become a commercially significant industry with vast potential and promising prospects for the future.

## CRediT authorship contribution statement

**Yuqi Ma:** Writing – review & editing, Writing – original draft, Visualization. **Xiaoyi Sun:** Writing – review & editing, Writing – original draft, Visualization. **Ziwei Cai:** Writing – review & editing. **Mengjing Tu:** Writing – review & editing. **Yugang Wang:** Writing – review & editing. **Qi Ouyang:** Writing – review & editing. **Xueqing Yan:** Writing – review & editing, Supervision, Conceptualization. **Gaoshan Jing:** Writing – review & editing, Supervision, Conceptualization. **Gen Yang:** Writing – review & editing, Supervision, Conceptualization.

## Declaration of competing interest

The authors declare that they have no known competing financial interests or personal relationships that could have appeared to influence the work reported in this paper.

## Data Availability

No data was used for the research described in the article.
